# Adsorption of Human Papillomavirus 16 to Live Human Sperm

**DOI:** 10.1371/journal.pone.0005847

**Published:** 2009-06-09

**Authors:** Julio Pérez-Andino, Christopher B. Buck, Katharina Ribbeck

**Affiliations:** 1 FAS Center for Systems Biology, Harvard University, Cambridge, Massachusetts, United States of America; 2 Laboratory of Cellular Oncology, National Cancer Institute, Bethesda, Maryland, United States of America; University of Sao Paulo, Brazil

## Abstract

Human Papillomaviruses (HPVs) are a diverse group of viruses that infect the skin and mucosal tissues of humans. A high-risk subgroup of HPVs is associated with virtually all cases of cervical cancer [1]–[3]. High-risk HPVs are transmitted sexually; however, the exact mechanisms by which sexual contact promotes virus infection remain uncertain. To study this question we asked whether capsids of HPV type 16 (a high-risk HPV) specifically interact with sperm cells. We tested if purified HPV16 virions directly adsorb to live human sperm cells in native semen and in conditions that resemble the female genital tract. We found that HPV16 capsids bind efficiently to two distinct sites at the equatorial region of the sperm head surface. Moreover, we observed that the interaction of virus with sperm can be reduced by two HPV infection inhibitors, heparin and carrageenan. Our findings suggest that 1) sperm cells may serve as motile carriers that promote virus dispersal and mucosal penetration, and 2) blocking interactions between HPV16 and sperm cells may be an important strategy for the development of antiviral therapies.

## Introduction

Human Papillomaviruses (HPVs) comprise a highly diverse group of non-enveloped DNA viruses. HPVs commonly infect mucosal genital epithelia, with an estimated 75% of humans being affected [1], [2]. Although many HPV infections remain clinically inconspicuous or develop benign symptoms such as warts (papillomas), a subset of infections lead to cancer of the uterine cervix [3]–[5]. The majority of cervical cancers are induced by one highly oncogenic HPV type, HPV16, which has received significant public attention for the past decade [3].

HPV16 is primarily transmitted through sexual intercourse. However, the exact mechanism by which sexual contact promotes virus infection remains unclear. To infect, the virus must overcome the body's first line of defense, a thick mucus layer in the female genital tract that shields the underlying cells from contact with noxious agents and pathogens [6]–[9]. We might expect that viruses have evolved strategies to facilitate their dispersal through the mucus gel. In cytoplasm, viruses exploit the motility of motor proteins [10]. In mucus, viruses perhaps also exploit exogenous sources of motility.

Sperm cells associated with sexual contact are highly motile and well adapted to passage through mucus [11]. We hypothesize that sexually transmitted viruses may have evolved to exploit sperm cells as vehicles for dispersal and mucus penetration within the female genital tract. If this hypothesis is correct, HPV16 should be able to bind to live human sperm cells.

## Results and Discussion

We aimed to test the possibility that HPVs can interact with sperm cells with an *in vitro* system. HPV16 is an excellent model to study this question because infectious HPV16 capsids, also called pseudovirions, can be produced *in vitro* in large quantities using cultured cell lines. Biochemical and genetic perturbations of HPV16 pseudovirions are therefore feasible; their interactions with defined mucus components and effects on virus motility can be studied directly. We used HPV16 capsids that were covalently labeled with Alexa Fluor 488 (Alexa488) for direct detection. This modification does not compromise capsid integrity or infectivity [12].

First, we studied HPV16-sperm interaction in freshly ejaculated, undiluted human semen. Fluorescent HPV16 capsids were added to semen at a final concentration of 80 µg/ml and the capsid-semen mixture was incubated at 37°C. At various time points, aliquots were taken and sperm were analyzed for association with HPV16 capsids by live fluorescence microscopy. Under these conditions, no HPV16 capsids were detected on the surface of sperm, even after several hours of incubation (data not shown).

Semen contains high concentrations of soluble proteins that could protect sperm from potentially deleterious interactions with virus capsids. When semen is passed into the female genital tract, it mixes with vaginal fluids. Seminal factors are diluted, and sperm are exposed to a comparably acidic environment, which can reach pH values below 4 due to lactic acid production by native vaginal microflora. Both the absence of seminal factors and low pH may affect the propensity of sperm cells to associate with HPV16.

**Figure 1:**
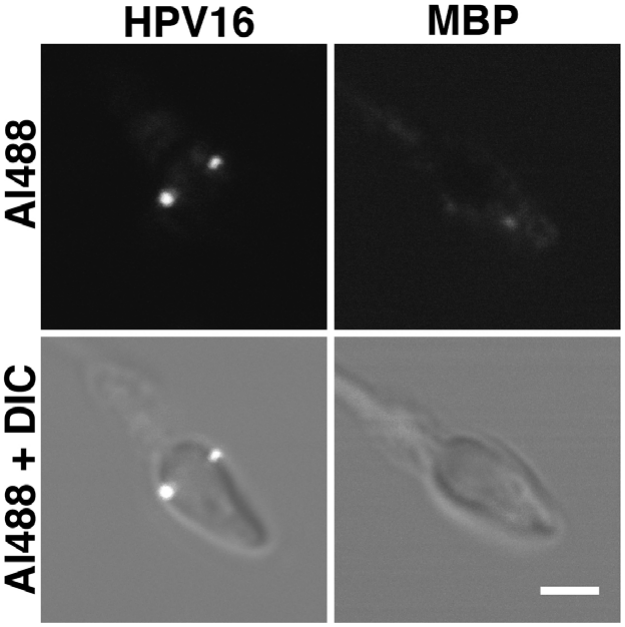
HPV16 capsids bind to distinct foci at the equatorial region of live sperm. Freshly ejaculated sperm cells were washed and subjected to purified Alexa488 labeled HPV16 capsids or Alexa488 maltose binding protein (MBP), which was used as a control. The protein-sperm reaction was incubated at 37°C for 10 min and subsequently analyzed by live confocal microscopy. The capsids accumulated specifically at two foci, one located on each side of the sperm head along its equator. It is crucial to note that this specific localization was detectable only on live sperm; on sperm with compromised viability, capsids stained a ring surrounding the entire sperm head. In contrast to HPV16 capsids, Alexa488 MBP bound faintly to irregular patches on sperm head and tail. Scale bar, 2 µm.

To test this possibility, we washed sperm prior to exposure with HPV16 capsids. To account for a possible influence of pH, we conducted the virus-sperm association reaction at pH 8.6, which resembles conditions in native semen, and pH 7.4. The washing procedure and the buffer composition were optimized to achieve maximal sperm viability for the duration of the experiment (see Material and Methods). In both conditions, HPV16 capsids rapidly adsorbed to the surface of the sperm heads. The capsids accumulated specifically at two foci, one located on each side of the sperm head along its equator (Fig. 1). Viral binding was observed on 52% of live sperm when the reaction was carried out at pH 8.6, and increased to 72% in more neutral conditions (pH 7.4) (Table 1).

**Table 1:** Quantification of HPV16-sperm interaction.

	Percentage sperm+/−SD associated with:	
	HPV16	MBP
native semen	0	0
*buffer*: pH 8.6	52.5+/−15.0	0
pH 7.4	71.6+/−2.0	0
+ι-carrageenan	33+/−17.5	0
+heparin	15.8+/−9.4	0

Freshly ejaculated sperm cells were washed and incubated with Alexa488 labeled HPV16 capsids or the control protein Alexa488 MBP. Sperm-washing and the virus-sperm binding reactions were carried out as indicated at pH 8.6 or pH 7.4 (for buffer composition see Methods). The virus-sperm mixture was incubated at 37°C for 10 min and subsequently analyzed by live microscopy. The number of sperm with fluorescent staining at one or both foci on the sperm head was determined. Only live sperm, i.e. visibly moving cells, were considered. The numbers represent averages drawn from three independent experiments. In each experiment, 50 sperm cells were evaluated. Note that the association of HPV16 to sperm increases in efficiency at neutral conditions. To assess the effect of carrageenan and heparin on the HPV16-sperm interaction, HPV16 capsids were pre-incubated with carrageenan (0.1 mg/ml) or heparin (0.1 mg/ml) at RT for 10 min to allow association of the molecules to the capsids. Thereafter, the capsid-inhibitor mixture was added to the washed sperm in buffer at pH 7.4. Both heparin and carrageenan significantly reduced binding of HPV16 to sperm.

Two controls assessed the specificity of the HPV16-sperm interaction: First, HPV16 capsids tagged with L2-GFP fusion protein [13] instead of Alexa488 also localized to the two foci (data not shown), confirming that the capsid, not the Alexa488 dye, mediated this interaction. Second, bacterially-produced Alexa488-labelled maltose binding protein (MBP) did not accumulate at these domains but instead bound weakly at irregular patches on sperm head and tail (Fig. 1 and Table 1). This suggests that certain properties specific to the HPV16 capsid, which are absent from MBP, facilitate binding to the foci on the sperm head.

Together, our data reveal that HPV16 capsids can bind live human sperm cells. Interactions of HPV with sperm have been observed previously [14]–[19]. Our study extends these observations in two important ways: it shows that HPV16 has two distinct binding sites along the equator of the sperm heads, and moreover, that virus association to these domains is more efficient in neutral conditions and after removal of soluble seminal factors. It is possible that seminal factors, when present at high concentrations, prevent interactions between HPV16 and sperm. We deduce that the association of HPV16 with sperm is unlikely to occur in native semen, but rather may be promoted in the female genital tract at comparably low pH following the dilution of seminal fluid.

A direct interaction with live sperm cells has been documented for the vertically transmitted fish rhabdovirus, which causes infectious hematopoietic necrosis in salmonids [20]. Salmonid fish are oviparous species with external fertilization. The rhabdovirus adsorbs to sperm and transfers with it to the egg through the open water. We propose that mechanistically similar interactions occur between HPV16 and human sperm, and that these interactions promote HPV16 dispersal and mucosal penetration in the female genital tract.

HPVs and numerous other sexually transmitted viruses initially bind to negatively charged cell-surface glycosaminoglycans (GAGs), particularly heparan sulfate proteoglycans (HSPGs), on cultured epithelial cells (reviewed in [21]). The surface of sperm is also densely coated with carbohydrates [22], which may provide viral binding sites similar to those found on epithelial surfaces. If this is correct, it should be possible to compete for virus attachment with soluble factors that resemble GAGs in chemical structure. Heparin and carrageenan, two sulfated polysaccharides, have this property. Both molecules directly bind to papillomavirus capsids and efficiently block their association with cell-surface HSPGs [12], [23]–[25]. Heparin is a highly sulfated form of heparan sulfate produced by mast cells. Carrageenan is a class of sulfated polysaccharide extracted from marine red algae (seaweed), which is used as a thickener in several commercially available sexual lubricant products.

Both HSPG-mimicking compounds significantly reduced binding of HPV16 to sperm (Table 1). This suggests that GAGs, or molecules of similar chemical structure, are present on specific domains of the sperm surface and enable attachment of HPV16. This interaction may be related to the biochemical mechanism by which HPV16 adsorbs to cell surface GAGs on epithelial cells at initial stages of infection. Importantly, many viruses other than HPV bind epithelial cell surface GAGs [21]. Two examples are human immunodeficiency virus (HIV) and herpes simplex virus (HSV), which are also mucosotropic viruses transmitted via sexual contact. Indeed, an association of HIV and HSV with sperm has been reported [26]–[33]. It is possible that these viruses bind to similar binding sites as HPV16, and that hitchhiking on sperm may be a general strategy used by sexually transmitted viruses to overcome long distances and mucus barriers in the female genital tract.

The potential importance of virus-sperm association for HPV16 dispersal and mucosal penetration suggests that blocking viruses from binding to sperm may be a new and general strategy to prevent sexually transmitted viral diseases. Peptides derived from HPV16 or benign intact viruses that shield binding sites on the surface of sperm are excellent candidates to achieve this. Such tools may offer an important advantage over vaccination with antigens. Viruses can rapidly evolve new surface properties to escape neutralization by specific antibodies. However, to bypass inhibitors of virus-sperm attachment, the virus must evolve new interaction mechanisms that exploit novel binding sites on the sperm head, which are presumably limited. Thus, blocking viral attachment to sperm may be a more sustainable method of disease prevention than conventional vaccination.

## Materials and Methods

### Reagents and buffers

Alexa488 labeled HPV16 capsids were produced as previously reported [12]. Detailed protocols for production and handling of HPV capsids are available at the website <http://home.ccr.cancer.gov/Lco/>. Heparin (H4784) and ι-carrageenan (C0414) were obtained from Sigma. Recombinant MBP was expressed, purified, and covalently labeled with Alexa488 as previously described [34].

### In vitro HPV16-sperm association reaction

For each experiment freshly ejaculated sperm from one of four donors was prepared. Semen was processed, and the experiments conducted, within 6 hours to maintain maximal sperm viability. To remove soluble seminal factors, native semen was diluted 1∶10 with reaction buffer (see below) at the desired pH and incubated for 10 min at 37°C. Sperm cells were pelleted at 300 rcf for 10 min, the supernatant was rapidly discarded, and the pelleted sperm cells were resuspended in prewarmed buffer to repeat the washing step. After three rounds of washing, sperm cells were resuspended in reaction buffer and used for incubation with the HPV16 capsids.

The reaction buffer for the virus-sperm association consisted of 50 mM Tris (pH 8.6 and 7.4), 35 mM NaCl, 3 mM KCl, 2 mM CaCl_2_, 1 mM MgSO_4_, 500 mM glucose, and 0.1% BSA. Similar effects were seen when Hepes instead of Tris was used as the buffering agent at pH 7.4.

Alexa488 labeled HPV16 capsids and Alexa488 MBP were added in separate reactions at final concentrations of 80 µg/mL and 5 µM, respectively. The reaction volume was 25 µL. To assess effects of carrageenan and heparin on the association of HPV16 with sperm, HPV16 capsids were pre-incubated with carrageenan or heparin at different concentrations at RT for 10 min to allow association of the molecules to the capsids. Thereafter, the inhibitor-HPV16 mixture was combined with washed sperm to start the reaction. The carrageenan and heparin concentrations that are indicated in Table 1 and in the main text were the concentrations at which maximal suppression of HPV16 attachment was observed in our experimental conditions.

Protein-sperm mixtures were incubated at 37°C. At different time points, aliquots were taken and live sperm were scored by fluorescence microscopy. Fifty live sperm cells were evaluated per experiment, and each experiment was repeated three times.
